# Dual Role of Phenyl Amides from Hempseed on BACE 1, PPARγ, and PGC-1α in N2a-APP Cells

**DOI:** 10.1021/acs.jnatprod.1c00435

**Published:** 2021-08-30

**Authors:** Julio Rea Martinez, Gordana Šelo, María Ángeles Fernández-Arche, Beatriz Bermudez, María Dolores García-Giménez

**Affiliations:** † Department of Process Engineering, Faculty of Food Technology, Josip Juraj Strossmayer University of Osijek, 31000 Osijek, Croatia; ‡ Department of Pharmacology, Faculty of Pharmacy, University of Seville, 41012 Sevilla, Spain; § Department of Cellular Biology, Faculty of Biology, University of Seville, 41012 Sevilla, Spain

## Abstract

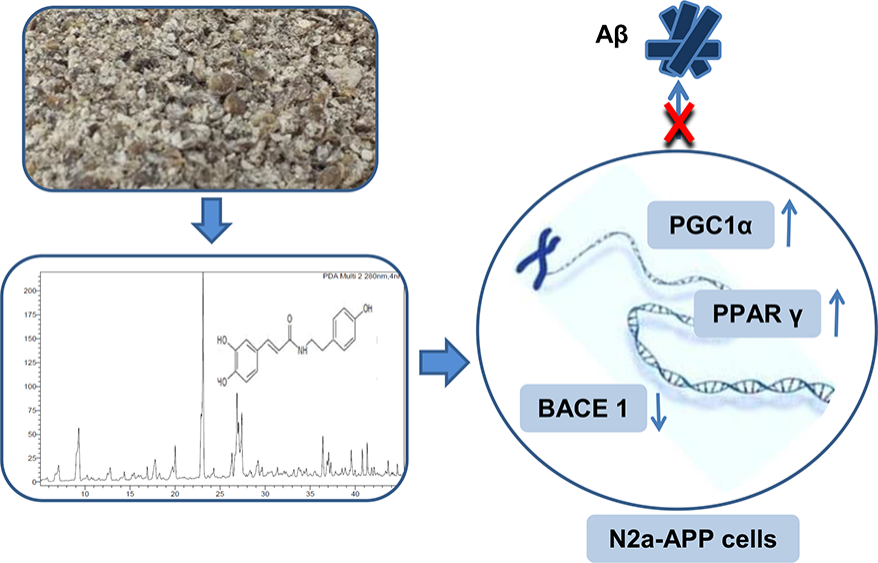

In Alzheimer’s disease (AD) the accumulation of amyloid β (Aβ) plaques in the brain leads to neuroinflammation, neuronal cell dysfunction, and progressive memory loss. Therefore, blocking the formation of Aβ plaques has emerged as one of the most promising strategies to develop AD treatments. Hempseed is widely used as a food, and recently its compounds have shown beneficial effects on neuroinflammation. The objective of this study was to investigate whether a fraction rich in phenyl amide compounds, *N*-*trans*-caffeoyltyramine (CAFT) and *N*-*trans*-coumaroyltyramine (CUMT), can affect gene expression: β-site amyloid-precursor-protein-cleaving enzyme 1 (BACE 1), peroxisome proliferator-activated receptor gamma (PPAR γ), and PPARγ-coactivator-1α (PGC-1α) in N2a-APP cells. The mRNA levels were measured using RT-qPCR. The ethyl acetate fraction and CAFT were found to reduce BACE1 gene expression and are promissory PPARγ and PGC-1α natural agonists. The results show that hempseed compounds can inhibit the expression of BACE 1, which is involved in the accumulation of Aβ plaques and positively affect transcription factors involved in complex and diverse biological functions.

Alzheimer’s disease is a neurodegenerative pathology characterized by the extracellular accumulation of amyloid β (Aβ) plaques, generated from amyloid β precursor protein (APP) via amyloidogenic pathways by β-secretase and γ-secretase and intracellular neurofibrillary tangles, and principally affects the elderly in terms progressive memory loss, cognitive damage, and deteriorating bodily functions. Although the etiology remains unclear, certain possible causes have been proposed, such as protein deposition (misfolding) disorders and aggregation of Aβ and tau proteins, activation of the innate immune system, mitochondrial dysfunction, and oxidative stress.^1,2^ With no possible treatment to control, prevent, or cure the devastating effects of this disease, therapy has focused only on treating the symptoms rather than understanding the pathology or other possible hallmarks.^3^ According to the latest report in 2019, it is estimated that over 50 million people worldwide suffer from dementia, and this number could increase to 152 million by 2050.^4^ In recent years, bioactive compounds from plants have shown promising effects in neurodegenerative diseases and appear to present an interesting source of alternative medicine for their evaluation regarding Alzheimer’s disease.^5,6^ Depending on their applicability, compounds should reach different areas of the central nervous system (CNS). The first step is to cross the blood–brain barrier (BBB), which uses anatomical, biochemical, and physicochemical mechanisms to control the exchange of different molecules between the blood and the brain.^7^ To solve this problem the parallel artificial membrane permeation assay (PAMPA-BBB) has been established as a predictive tool for the early stages of the discovery of drugs, which filters the possible compounds from natural sources or plant extracts that can penetrate the BBB.^8^

Hempseed from *Cannabis sativa* L. or similar is a well-known seed that has traditionally been used as both a food and a medicine and provides a source of high concentrations of polyunsaturated fatty acids, proteins, and vitamins.^9,10^ Recently, positive effects of its compounds on neuroinflammation and memory dysfunction have been reported.^11−13^ Previously, acetylcholinesterase inhibitory activities and beneficial effects from hempseed compounds were described in degenerative processes associated with inflammation and oxidative stress. However, whether the ethyl acetate fraction and isolated compounds from hempseed play a role in inhibition of BACE 1, which is involved in Aβ formation, a neuropathological feature associated with the early stages of Alzheimer’s disease (AD),^14^ and two negative regulators of BACE 1 in the form of PGC-1α, which regulate the transcription of BACE 1, and further Aβ formation in AD,^3^ and in the form of PPAR γ, which is involved in the regulation of the transcription of genes for anti-inflammation, redox homeostasis, glucose and lipid metabolism, and tissue recovery of acute brain injuries, among others,^15^ has yet to be studied and is an attractive target in numerous therapies for neurological disorders. In the search for natural BACE 1 inhibitors, we focus on a promising fraction, the ethyl acetate fraction (EAF), which has been obtained from defatted hempseed with a high content of phenyl amides and include two isolated compounds (caffeoyltyramine and coumaroyltyramine). In order to evaluate the possible inhibitory effects on BACE 1 and postproduction APP, the fraction and compounds were assessed *in vitro*, using mutant APP-overexpressed N2a cells.

## Results and Discussion

### Phytochemical Results

**Figure 1:**
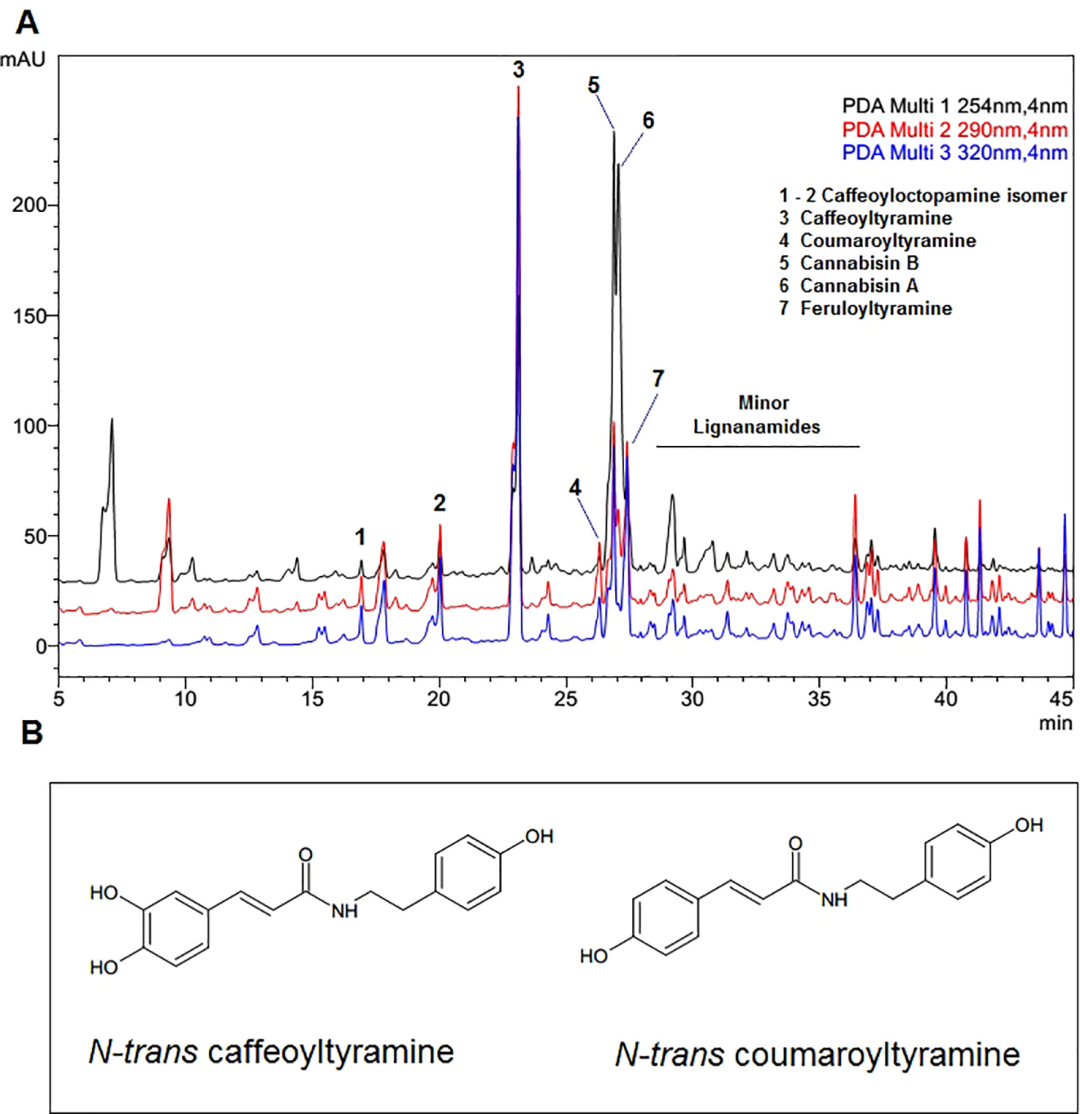
(A) UHPLC profile of the ethyl acetate fraction from hempseed from *Cannabis sativa* L. (B) Structures of *N-trans*-caffeoyltyramine and *N-trans*-coumaroyltyramine.

The chromatographic profile of the ethyl acetate fraction and two phenyl amides is shown in Figure 1. Analysis was performed by ultra-high-performance liquid chromatography (UHPLC) as described by Bucić-Kojić et al.^16^ The majority of the ethyl acetate fraction consists of phenyl amide compounds and, in smaller amounts, contains acid phenols, flavonoids, and terpenphenols. Studies have shown that caffeoyltyramine is the major compound in the fraction with a concentration above 6.36 mg/g extract.^17^

### Qualitative PAMPA-BBB Assay of the EAF

**Figure 2:**
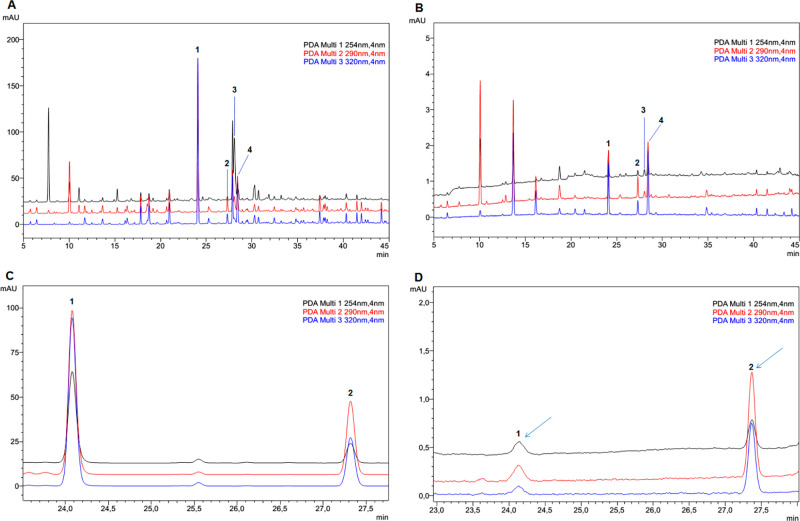
Chromatograms of EAF: (A) Stock solution in donor solution before and (B) acceptor solution after the PAMPA-BBB assay. Isolated compounds: (C) Stock solution of isolated compounds in donor solution before and (D) acceptor solution after assay. Phenyl amides: (1) *N-trans*-caffeoyltyramine, *t*_R_ 24.08 min; (2) *N-trans*-coumaroyltyramine, *t*_R_ 27.32 min; (3) cannabisin A, *t*_R_ 28.07 min; (4) feruloyltyramine, *t*_R_ 28.42 min, and lignanamide were identified in the acceptor solution after assays.

PAMPA-BBB assays constitute one of the most widely used models for the prediction of transcellular passive absorption *in vitro*, by filtering out potential compounds with applications in the CNS early in their development. In order to ascertain the applicability of the PAMPA-BBB system, the EAF was screened. The initial solution (donor) containing possible brain-permeable compounds was screened through a model of a lipid-infused artificial membrane on a solid filter support. This was analyzed by means of the UHPLC method described,^16^ as well as the acceptor solution compartment after the permeability assay, whose solution contains certain constituents of the donor (Figure 2). Although fractions/extracts rich in phenyl amides from hempseed have shown antineuroinflammatory^13^ effects, increased biogenic amine levels in mice striatum^18^ in animal models, and inhibition of U-87 cancer cell proliferation *in vitro*,^19^ it has also been shown that isolated compounds exert antineuroinflammatory effects on lipopolysaccharide (LPS)-induced BV2 microglia cells.^11^ However, no data on hempseed compounds indicate that it can cross the BBB and reach different areas of the brain. After EAF assessment, phenyl amides such as *N-trans*-caffeoyltyramine, *N-trans*-coumaroyltyramine (Figure 2D), feruloyltyramine, and the lignanamide cannabisin A were detected in the acceptor solution (Figure 2B). These compounds derive from the products of conjugation between phenolic acids and arylmonoamines, such as tyramine and octopamine, which are present in hempseed in large proportions^19,20^ and in high quantities.^17^ Hempseed can be a source of potential candidates with application in neurodegenerative diseases. It has also been reported that *N*-methylated tyramine derivatives of *Ginkgo biloba* can cross the BBB.^21^ Although the permeability assay is a filtering tool for the selection of potential brain-permeable compounds from plant extracts, *in vivo* assays and specific studies are required for the identification of compounds that can cross the BBB.

#### Effect of the EAF and Phenyl Amides on BACE 1 Gene Expression in N2a-APP Cells

**Figure 3:**
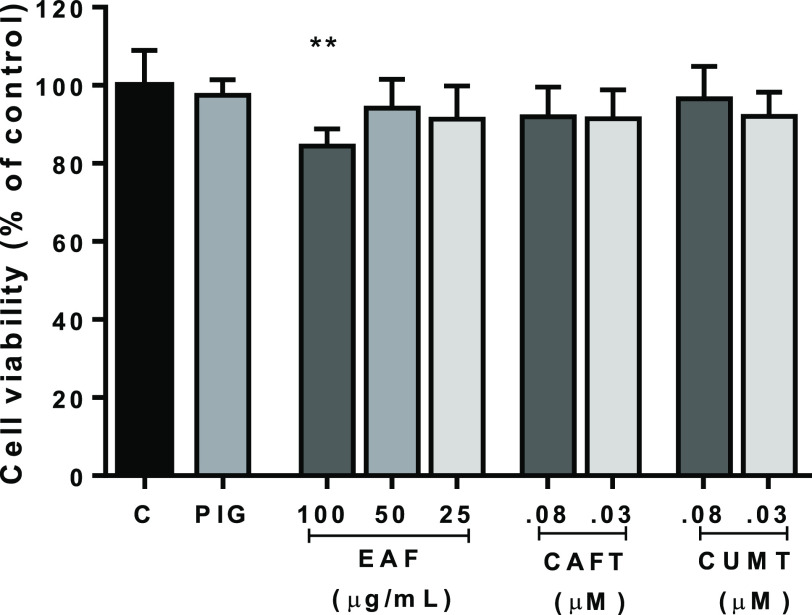
N2a-APP cells were treated with the ethyl acetate fraction (EAF) at 100, 50, and 25 μg/mL, compounds *N-trans*-caffeoyltyramine (CAFT) and *N-trans*-coumaroyltyramine (CUMT) at 0.03–0.08 μM, and PIG (14 μM) as positive control for 24 h, respectively. Cell viability was detected by the MTT assay. The results showed that the EAF at high dose had a significant effect on cell viability. All data are presented as means ± SD. The *p* values were calculated using one-way ANOVA, **p < 0.01 vs control group.

Results have shown that polar fractions from defatted hempseed present a strong radical scavenging activity and anti-inflammatory effect in human primary monocytes. Moreover, quantification shows a high content of phenyl amide compounds in the fraction composition in comparison with other phenols and flavonoids.^17^ The addition of ultrasonic treatment and temperature (45 °C) in the extraction process has increased the content of phenyl amide compounds. The data are similar to those reported in the literature where the application of heat during ultrasonic extraction treatment improved the yield of polyphenol content in extracts from seed cake powder.^22^ It has been reported that an extract rich in phenyl amides improves cognitive functions and reduces the expression of pro-inflammatory cytokines in the brain of LPS-induced mice at a concentration of 1 g/kg^13^ and significantly reduces TNF-α expression in BV2 microglial cells.^11^ However, for N2a-APP cells, the protection and cytotoxicity of the ethyl acetate fraction and *N-trans*-caffeoyltyramine (CAFT) and *N-trans*-coumaroyltyramine (CUMT) derivatives have yet to be evaluated. In this study, the EAF with a high content of phenyl amide compounds at concentrations of 25–100 μg/mL and the isolated compounds (CAFT and CUMT) at 0.03–0.08 μM were selected to treat N2a-APP cells for 24 and 48 h, respectively. The MTT assay showed that only the EAF fraction at high concentrations affected cell viability. The compounds had no effect on cell viability at the concentrations tested (Figure 3).

**Figure 4:**
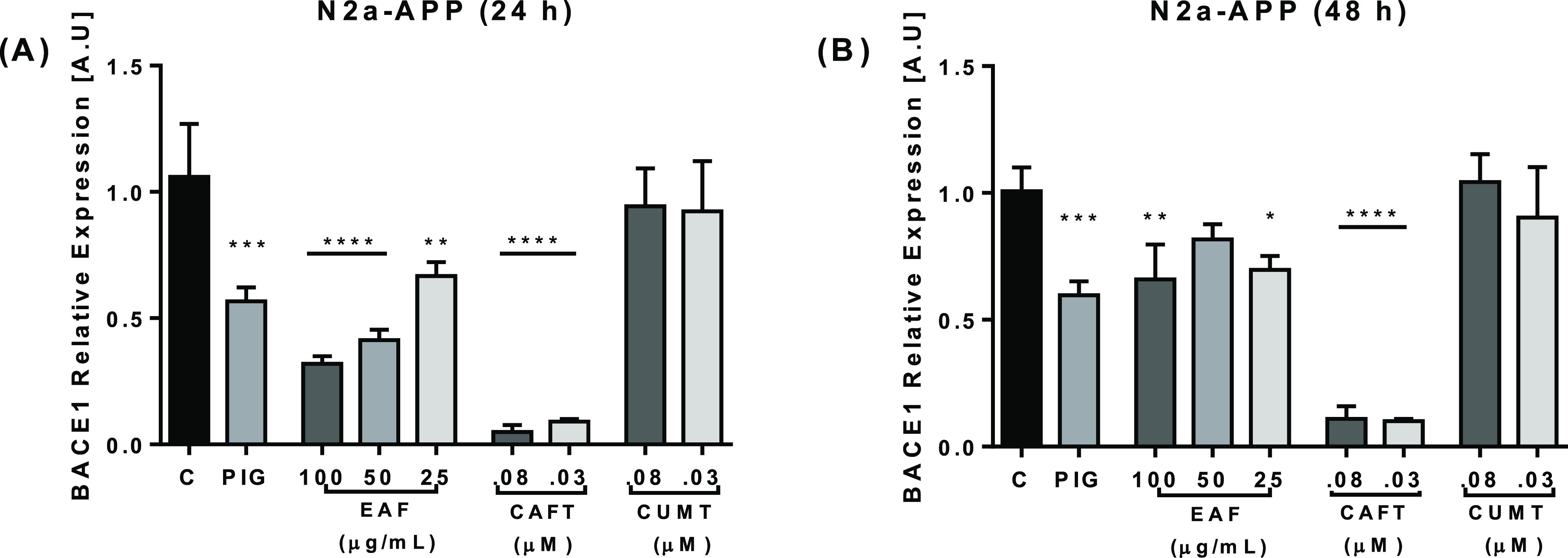
Effects of the EAF, CAFT, and CUMT on BACE 1 gene expression. N2a-APP cells were treated with the EAF at 25–100 μg/mL and CAFT and CUMT at 0.03–0.08 μM for 24 h (A) and 48 h (B). PIG (14 μM) was used as positive control. The result showed significant reduction in BACE 1 gene expression of the EAF and CAFT after 24 h (A), and after 48 h of treatment the EAF decreased activity on gene expression, CAFT showed stable activity suppressing gene expression (B), and CUMT showed no significant changes. EAF-, CAFT-, and CUMT-treated cells were compared with control cells. Data are expressed as mean ± SD, *n* = 3. The *p* values were calculated using one-way ANOVA, *p < 0.05; **p < 0.01; ***p < 0.001; ****p < 0.0001.

Using this culture system, we first analyzed whether EAF treatment and the two phenyl amide compounds could inhibit BACE 1 expression. After 24 h of treatment, our results showed a strong inhibition of BACE 1 expression, with significant differences in the EAF (μg/mL) and caffeoyltyramine (μM) at all tested concentrations, compared to the control cells (Figure 4). However, after 48 h of treatment, the EAF was less effective in reducing gene expression, while CAFT was shown to remain active and to suppress BACE 1 gene expression. Pioglitazone (PIG), a special pharmacological PPARγ agonist used in studies to elucidate the neuroprotective mechanism, was used as the positive control.^23^ The enzyme BACE 1 is necessary for the formation of all monomeric forms of Aβ peptides. Its subsequent accumulation in vulnerable brain parts is linked to the main cause of Alzheimer’s disease pathogenesis.^24,25^ In recent years, and with evidence supporting the amyloid hypothesis as the main factor responsible for the initiation of AD, large amounts of resources have been devoted to the search for potential drug candidates that can act as BACE 1 inhibitors.^26^ Furthermore, more and more research is focusing on the use of medicinal plants as a promising source of molecules against AD.^27^ These results show that EAF rich in phenyl amides and the main compound present in the fraction (caffeoyltyramine) have an inhibitory effect on the expression of BACE 1. The inhibitory result may help to interrupt Aβ generation and accumulation, which play a role the development of the pathology of Alzheimer’s disease. Studies consider metabolites of plants to be a useful platform in the discovery and development of drugs for the treatment of AD, and phenylpropanoid metabolites have emerged as prime candidates due their diverse biological functions.^2^ Caffeoyltyramine has shown protective effects against H_2_O_2_-induced neurotoxicity in PC12 cells in other studies and antineuroinflammatory activity by down-regulating TNF-α released by LPS-induced BV2 cells.^11,28^ This indicates that *N-trans*-caffeoyltyramine may suppress the production and the secretion of Aβ by means of the inhibition of the enzyme involved in its generation.

#### Effect of EAF and Phenyl Amides on PPARγ Gene Expression in N2a-APP Cells

**Figure 5:**
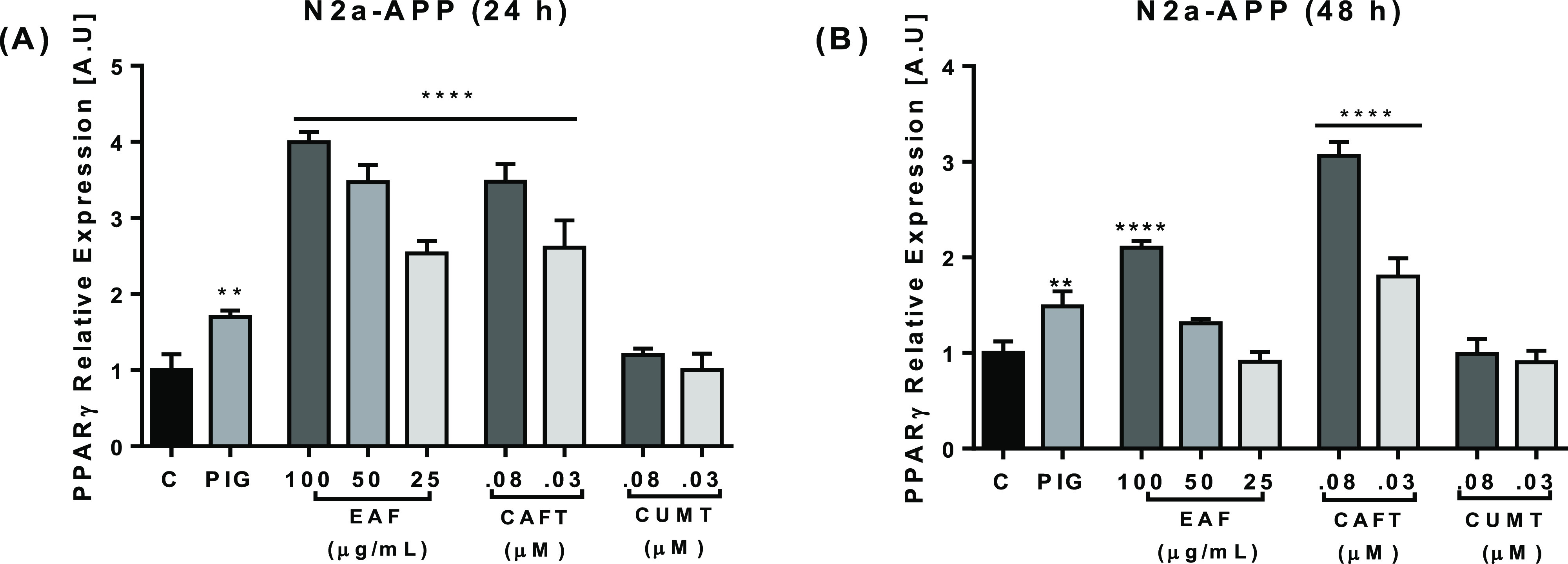
EAF, CAFT, and CUMT promoted PPARγ gene expression. N2a-APP cells were treated with the EAF at 25–100 μg/mL and CAFT and CUMT at 0.03–0.08 μM for 24 h (A) and 48 h (B). PIG (14 μM) was used as positive control. The result showed significant changes in PPARγ gene expression of the EAF and CAFT after 24 h at all concentrations tested (A), and after 48 h of treatment EAF and CAFT showed decreasing activity (B) and CUMT showed no significant changes. EAF-, CAFT-, and CUMT-treated cells were compared with control cells. Data are expressed as mean ± SD, *n* = 3. The *p* values were calculated using one-way ANOVA, *p < 0.05; **p < 0.01; ***p < 0.001; ****p < 0.0001.

In order to further confirm whether defatted hempseed compounds up-regulate PPARγ expression in N2a-APP cells, the gene expression was measured of cells treated with the EAF and its metabolites for 24 and 48 h. The result showed a significant increase in PPARγ gene expression by the EAF in cells treated for 24 h at all concentrations in the evaluation. Although the expression decreased after 48 h of treatment, differences with the control cells remained only in high EAF concentrations (Figure 5). However, caffeoyltyramine showed a substantially higher PPARγ expression compared to that of total EAF with a slight decrease after 48 h of treatment (Figure 5B). As described above (BACE 1), CAFT retained its activity over time and induced PPARγ gene expression. Coumaroyltyramine showed no effect on the PPARγ expression. Studies have shown that PPARγ regulates the transcription of genes involved in lipid and glucose metabolism, inflammation, and redox equilibrium, among others. The overexpression of BACE 1 in the brain has been observed under inflammatory conditions, activated by pro-inflammatory cytokines, such as interleukin-1β (IL-1β), IL-6, and tumor necrosis factor-α (TNF-α) released by microglial cells. PPARγ is also affected by the secretion of inflammatory cytokines, thereby strongly reducing their expression, an effect that can be suppressed with the use of PPARγ agonist drugs, which have emerged as a new therapy in the treatment of AD. The regulation of the transcription of the enzyme BACE 1 responsible for the production of neurotoxic amyloid β oligomers seems to be the principal therapeutic target of AD drug production, and reports show how PPARγ regulates their transcription.^15,29,30^ Results show that EAF and CAFT treatment could reduce Aβ deposition in the brain by means of decreased levels of BACE 1 gene expression through the activation of the PPAR γ pathway, which is a well-known regulator of BACE 1.

#### Effect of Hempseed EAF and Phenyl Amides on PPARγ Coactivator-1α (PGC-1α) Gene Expression

**Figure 6:**
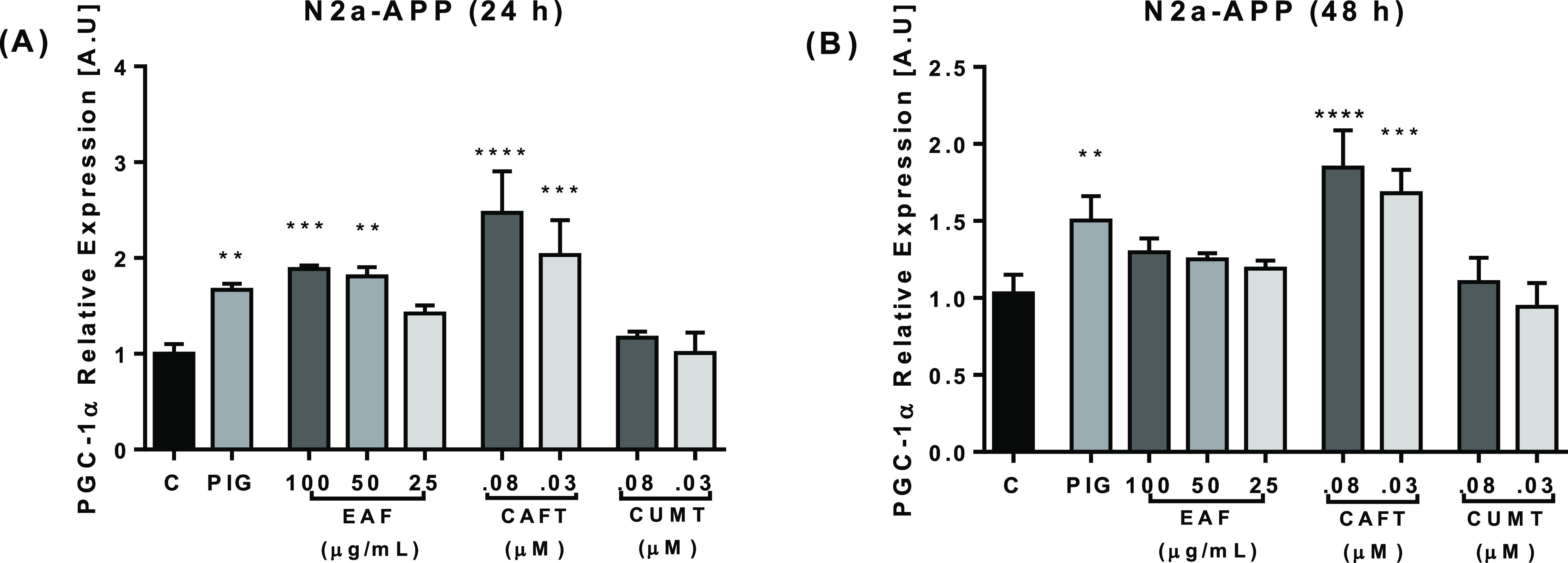
EAF, CAFT, and CUMT promoted PGC-1α gene expression. N2a APP cells were treated with the EAF at 25–100 μg/mL and CAFT and CUMT at 0.03–0.08 μM for 24 h (A) and 48 h (B). PIG (14 μM) was used as positive control. Gene expression levels showed significant changes of PGC-1α in the EAF and CAFT after 24 h (A), and after 48 h treatment, only the expression levels of CAFT were markedly increased in treated cells (B) and CUMT showed no significant changes. EAF-, CAFT-, and CUMT-treated cells were compared with control cells. Data are expressed as mean ± SD, *n* = 3. The *p* values were calculated using one-way ANOVA, *p < 0.05; **p < 0.01; ***p < 0.001; ****p < 0.0001.

EAF treatment for 24 h significantly increased PGC-1α expression. On the other hand, treatment for 48 h has a tendency to decrease said expression and shows no differences with control cells in gene expression. Similar results are found for caffeoyltyramine in both 24 and 48 h treatments with significant differences in relation with the control, while coumaroyltyramine shows no effects on PGC-1α expression (Figure 6). Our present study demonstrates that incubation with CAFT for 24 and 48 h results in an apparent up-regulating of PGC-1α gene expression in N2a-APP cells. PGC-1α is a coactivator involved in the transcription of PPARγ and the regulation of mitochondrial biogenesis, fatty acids, respiratory capacity, and oxidative metabolism.^3,31,32^ Studies point to its participation in neurodegenerative diseases, where decreased mRNA expression in the brain with Alzheimer’s pathology has been found.^33^ Overall, caffeoyltyramine may have negatively regulated BACE 1 activity (Figure 4). Various inflammation-related transcription factors, such as PPARγ, NF-κB, and PGC-1α, are involved in the process of BACE 1 regulation. Given that PGC-1α plays a role in PPARγ transcription and that both are associated with neurodegenerative disorders, the positive effect of EAF and caffeoyltyramine on the expression of these two genes could lead to a positive impact in the prevention of mental decline and could work as neuroprotective agents. However, further studies are needed to determine the pathways related to the transcription of these negative regulators of BACE 1. This study reveals the beneficial effects of the ethyl acetate fraction and main compound *N-trans* caffeoyltyramine from hempseed in the prevention as well as in the potential treatment of neurodegenerative diseases, through the positive effect on hallmarks involved in the development of Alzheimer’s diseases by reducing BACE 1 and increasing PGC-1α and PPARγ gene expression. In this context, hempseed constitutes a rich source of various bioactive compounds. Consumption of the ethyl acetate fraction and its bioactive metabolites has shown a wide range of promising activities with various human health benefits, and this report indicates the neuroprotective properties of this vegetal. In the future, additional molecular studies together with clinical trials are required to establish the therapeutic safety and efficacy of EAF and CAFT.

## Experimental Section

### Chemical and Reagents

3-(4,5-Dimethylthiazol-2-yl)-2,5-iphenyltetrazolium bromide (MTT), dimethyl sulfoxide (DMSO), *n*-dodecane, cholesterol, TRI reagent, pioglitazone, *N-trans*-caffeoyltyramine, and *N-trans*-coumaroyltyramine were purchased from Sigma-Aldrich, iScript cDNA synthesis kit was from BIO-RAD, methanol and formic acid LC-MS grade were from VWR Chemicals, DMEM medium was from Biochrom AG, and brain polar lipid extract was from Avanti Polar Lipids.

### Plant Material

The hempseed (Jarad-Seed; batch: 250816) was acquired in the province of Seville, Spain.

### Preparation of the Fraction Tested

An ethyl acetate fraction and two phenolic amides isolated from it were used for our tests, and the procedure is described below: Hempseeds (3 kg) were crushed and defatted three times with *n*-hexane (each for 24 h). After filtration and evaporation of the *n*-hexane, the defatted seeds were extracted with 75% aqueous ethanol, solvent in a ratio 1:3, twice (each for 24 h), followed by ultrasonic bath extraction (Ultrasons HD, JP Selecta) (solvent in a ratio 1:1) with a fixed power (180 W) for 20 min at 45 °C with periodic stirring. The filtrates were concentrated under vacuum until the volume was reduced to about 500 mL and were stored in 100 mL tubes at 4 °C for 48 h. Then, this aqueous solution was liquid–liquid extracted with ethyl acetate (4 × 500 mL), and the ethyl acetate solution was subsequently evaporated under vacuum, freeze-dried, and stored in a dark glass bottle at 4 °C prior to analysis, resulting in the EAF.

### Compound Isolation

Isolation was performed as previously reported^18^ from the ethyl acetate fraction (4.3 g), fractionated by column chromatography with approximately 85.0 g of silica gel (1:20 ratio). The following solvent mixtures were used, in a volume of 500 mL each: hexane–ethyl acetate (80:20–0:100) and ethyl acetate–methanol (80:20–0:100). The tubes were pooled together according to their similarity in thin-layer chromatography. The compounds from the hexane–ethyl acetate (20:80–0:100) fractions were recovered and purified with Sephadex LH-20 using methanol. The isolated compounds were analyzed and confirmed by the UHPLC HRMS/MS method,^17^ retention time, MS data, fragmentation, and UV spectrum and compared with the corresponding standards. The purity of isolated compounds was >90%; 9 mg of *N-trans*-coumaroyltyramine and 38 mg of *N-trans*-caffeoyltyramine were obtained.

### Qualitative PAMPA-BBB Procedure

PAMPA was used as a high-throughput assay to predict the BBB permeation of the isolated compounds and the total fraction, following the process detailed in the bibliography with slight modifications.^34^ Stock solutions of the isolated compounds (2 mg/mL) and the EAF (20 mg/mL) were diluted in ETOH 50%, filtered with a 0.45 μm pore size, and mixed with phosphate-buffered saline (0.01 M PBS, pH 7.4) to obtain a donor start solution with a final concentration of 200 μg/mL for the compounds and 2 mg/mL for the EAF, respectively. The filter membrane of the donor (top) plate (96-well polycarbonate-based filter plate, Multiscreen-IP, MAIPN4510, pore size 0.45 μm, Millipore) was coated with 5 μL of BBB-specific lipid solution (16 mg of PBL and 8 mg of cholesterol dissolved in 600 μL of *n*-dodecane), and the well acceptor plate (bottom) was filled with 300 μL of PBS buffer. Then, a 150 μL aliquot of the samples was applied to a donor well and carefully placed on the acceptor plate to form a ¨sandwicḧ and left undisturbed for 4 h at 37 °C. After incubation, the acceptor plate was separated from the donor plate. EAF compounds in the donor starting solution and in both donor and acceptor wells after the incubation period underwent UHPLC (Nexera XR, Shimadzu, Japan) in triplicate with UV detection from 200 to 400 nm according to the described method.^16^ Chromatograms were extracted at the appropriate wavelengths.

### Cell Culture and Treatment

Mutant APP-overexpressed N2a (N2a-APP) cells were used for the study. Cells were cultured in Dulbecco’s modified Eagle’s medium (DMEM) containing 10% fetal bovine serum (FBS) and l-glutamine in 5% CO_2_ at 37 °C. Cells were plated in six-well plates at a density of 5 × 10^5^ /mL for 24 h and 5 × 10^5^/2 mL for 48 h of treatment with the ethyl acetate fraction at 25–100 μg/mL, compounds at 0.03–0.08 μM, and the PPARγ agonist pioglitazone (14 μM). Control cells were incubated with medium alone.

### Cell Viability Assay (MTT)

N2a-APP cells were incubated in a 96-well plate (1 × 10^4^ cells/well) for 24 h with various concentrations of 25–100 μg/mL of ethyl acetate fraction and 0.03–0.08 μM *N-trans*-caffeoyltyramine and -coumaroyltyramine. Cell control were incubated with medium alone. Afterward, cells were incubated with MTT (1 mg/mL) for 2 h at 37 °C until a purple precipitate was visible. MTT-formazan crystals were solubilized with DMSO (200 μL) and then measured with a microplate reader at 570 nm corrected to 650 nm. Cell survival was expressed as a percentage of absorbance compared with nontreated cells.

### RNA Isolation and RT-qPCR Analysis

After the incubation period, total RNA was extracted using TRI Reagent (Sigma) as indicated by the manufacturer. The A260/A280 ratio in a NanoDrop ND-1000 spectrophotometer (Thermo Scientific, Madrid, Spain) was used to determine RNA quality. Momentarily, RNA (1 μg) was subjected to reverse transcription (iScript, Bio-Rad, Madrid, Spain) according to the manufacturer’s protocol. A 20 ng amount of the resulting cDNA was used as a template for real-time PCR amplifications. The mRNA levels for specific genes were determined in an MX3000P system (Stratagene). For each PCR reaction, the cDNA template was added to Brilliant SYBR green QPCR Supermix (Bio-Rad), which contained primer pairs for either gene or glyceraldehyde 3-phosphate dehydrogenase (GAPDH) and m18s as housekeeping genes (Table 1). All amplification reactions were performed in triplicates, and the average threshold cycle (Ct) counts of the triplicates were used to calculate the relative mRNA expression of candidate genes. The magnitude of change in mRNA expression for the candidate genes was calculated using the standard 2^–(ΔΔCt)^ method. All data were normalized to the levels of the endogenous reference genes (GAPDH and 18s) and expressed as a percentage of controls.

**Table 1:** Sequences of RT-qPCR Primers for Gene Expression Analysis

target	GenBank accession number	direction	sequence (5′→3′)
mBACE1	AF190726.2	forward	AGAGGCAGCTTTGTGGAGAT
		reverse	CTGGTAGTAGCGATGCAGGA
mPGC1	NM_008904.2	forward	AGCCTCTTTGCCCAGATCTT
		reverse	GGCAATCCGTCTTCATCCAC
mPPARg	NM_001127330.2	forward	AGGGCGATCTTGACAGGAAA
		reverse	CGAAACTGGCACCCTTGAAA
mGAPDH	NM_008084.3	forward	CAACTCCCACTCTTCCACCT
		reverse	GAGTTGGGATAGGGCCTCTC
m18s	NM_008084.3	forward	AGAAACGGCTACCACATCCA
		reverse	CCCTCCAATGGATCCTCGTT

### Statistical Analysis

All values are expressed as arithmetic means ± standard deviations (SD). Data were evaluated using Graph Pad Prism version 6.01 software (San Diego, CA, USA). Statistical significance of differences between each parameter in the groups was evaluated using one-way analysis of variance (ANOVA), followed by Dunnett’s multiple comparisons test as a *post hoc* test. *p*-Values less than 0.05 were considered statistically significant
